# Non-Operative Management of a Pediatric Patient With Bilateral Subdural Hematomas in the Setting of Ruptured Arachnoid Cyst

**DOI:** 10.7759/cureus.20099

**Published:** 2021-12-02

**Authors:** Boyi Li, Christina Ng, Eric Feldstein, Carrie Muh, Avinash Mohan, Michael Tobias

**Affiliations:** 1 Neurological Surgery, University of North Carolina at Chapel Hill School of Medicine, Valhalla, USA; 2 Neurological Surgery, Westchester Medical Center, Valhalla, USA

**Keywords:** intracranial hypotension, middle cranial fossa, bilateral, subdural hematoma, arachnoid cyst

## Abstract

Pediatric subdural hematomas (SDH) are associated with arachnoid cysts (AC), particularly in the middle cranial fossa (MCF). Operative management of these hemorrhages is a mainstay of treatment. Conservative management may be an option if there is minimal mass effect and the patient is mildly symptomatic. A 14-year-old male presented with right frontal headaches that worsened with activity. He was found to have a large right MCF AC. Scheduled routine outpatient follow-up CT of the head demonstrated bilateral SDH. There was no history of significant head trauma. He was admitted for close observation and his inpatient scans remained stable. Outpatient follow-up imaging over the course of three and a half years demonstrated resolution of SDH and decreased AC size. He denied headaches and continued doing well in school. ACs are a risk factor for the development of SDH in young male patients after minor trauma. Development of intracranial hypotension secondary to AC rupture may have contributed to the development of bilateral SDH in our patient. We demonstrate here that close clinical follow up with serial imaging may be considered a management strategy in these patients.

## Introduction

Arachnoid cysts (AC) are cerebrospinal fluid (CSF) filled spaces that are non-neoplastic, with the majority of them (50%-65%) found in the middle cranial fossa (MCF) [1-3]. In the MCF, the Galassi Classification classifies AC based on size, displacement of adjacent structures, and midline shift [4]. The prevalence of AC in the population is estimated to be 1.4%-1.6% based on incidental MRI findings of middle-aged adults, and up to 2.6% on pediatric brain MRI [2,5,6].

Symptoms are thought to develop secondary to enlargement of the AC, with younger age at presentation, larger size of the cyst, and presence of cyst-associated symptoms being predictors for surgical management in pediatric cases [7,8]. The natural history in pediatric AC is overwhelmingly benign but can be associated with headache, hydrocephalus, seizures, cognitive sequela, and rupture into the subdural space [7,9]. Repeat imaging in six months to one year to ensure stability is frequently considered appropriate, although no standardized surveillance protocol exists [9].

In cases where symptoms are attributable to the AC, surgery is a reasonable option [3]. However, the small risk of associated subdural hematoma (SDH) does not alone justify treatment of the otherwise stable, asymptomatic AC [7].

## Case presentation

**Figure 1:**
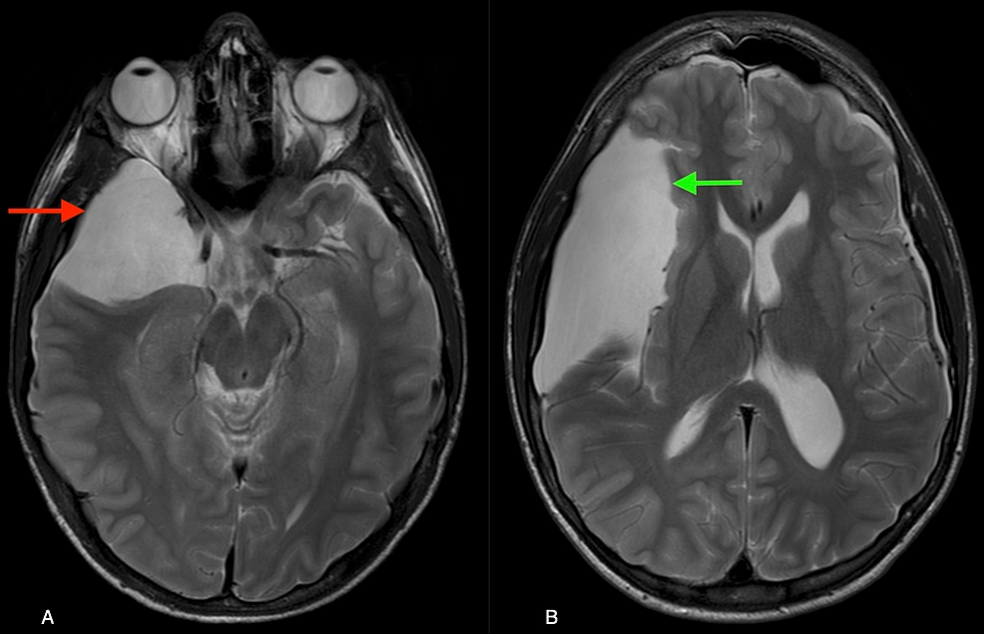
Axial T2 MRI brain at the level of the midbrain (A) and basal ganglia (B) demonstrating a large right middle fossa arachnoid cyst (red arrow) extending into the right sylvian fissure (green arrow).

A 14-year-old male with a history of asthma presented to the emergency room with isolated right frontal headaches that became worse with activity. There was no history of recent trauma and review of systems was otherwise negative. The patient was neurologically intact. An MRI of the brain demonstrated a large right MCF AC (Figure 1). Neurology consult recommended conservative management of his headache. Ophthalmological exam revealed no papilledema and the patient was discharged with follow up imaging planned several weeks later.

**Figure 2:**
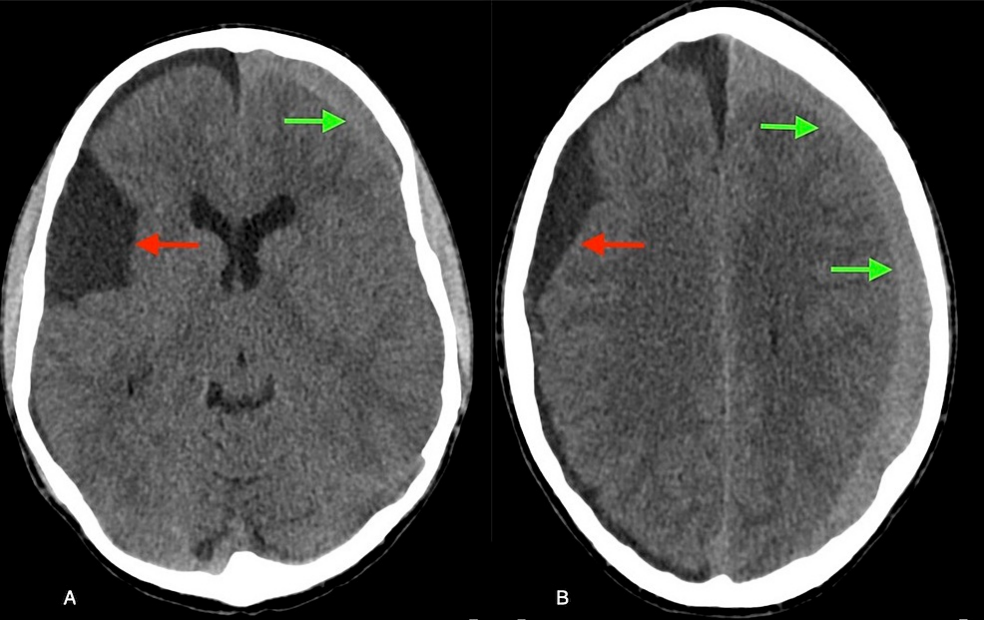
Axial CT scan of the head without contrast showing a new right chronic appearing subdural hematoma (SDH) (red arrows) and acute left SDH (green arrows).

**Figure 3:**
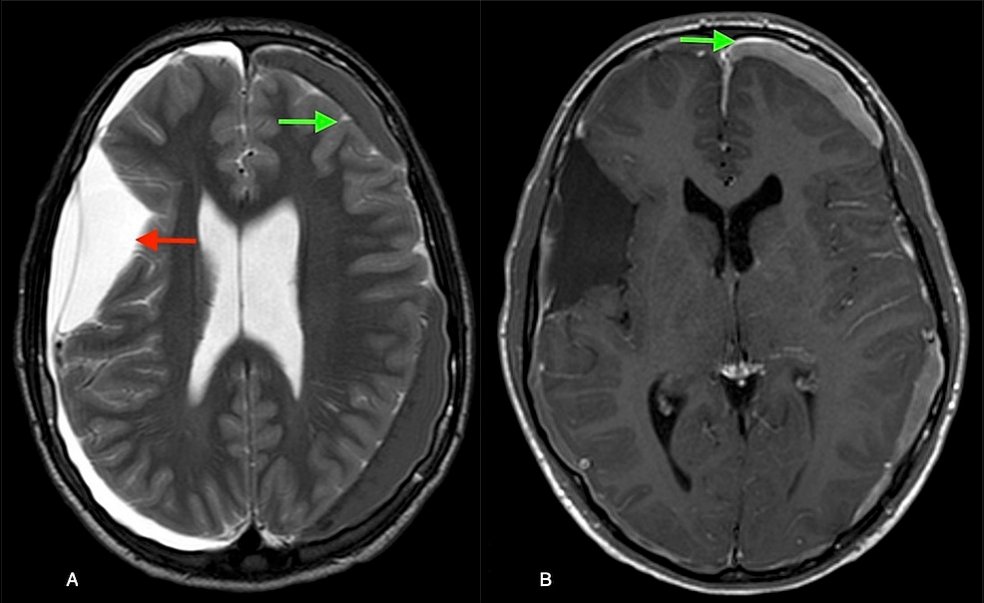
Axial T2 MRI brain (3A) showing decreased arachnoid cyst (red arrow) and subdural hematoma (green arrow). Contrast-enhanced axial sequence (3B) showing pachymeningeal enhancement (green arrow).

On his routine follow-up CT head, a new right chronic SDH and a new left acute SDH was found (Figure 2). He was brought in to the emergency room for evaluation where he was found to be neurologically intact but endorsed two recent episodes of morning emesis. Hematological workup was negative for bleeding disorders. Contrast-enhanced brain MRI revealed pachymeningeal enhancement and decreased AC size (Figure 3).

**Figure 4:**
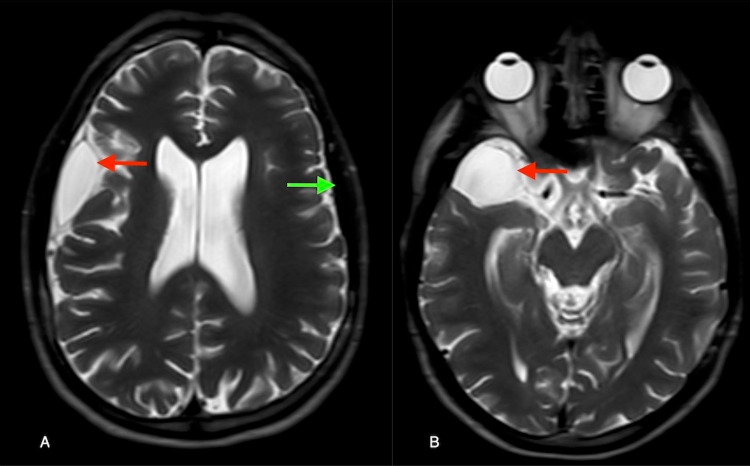
Axial T2 MRI brain at the level of the lateral ventricles (A) and the midbrain (B) demonstrating interval resolution of acute subdural hematoma (SDH) (green arrow) and chronic SDH (red arrow) and arachnoid cyst (red arrow) at 3.5 year follow-up.

Serial imaging remained stable and he was discharged with plans for repeat imaging. As of 3.5 years later, the patient is doing excellent clinically and his MRI brain demonstrates resolution of bilateral SDH and decreased size of the AC (Figure 4). The patient remains asymptomatic and is doing well in school.

## Discussion

Chronic SDHs are commonly encountered in adults but are rare in the pediatric population [10,11]. In the pediatric population, chronic SDH is associated with AC [12]. Particularly, cyst location in the MCF is recognized for being associated with increased susceptibility to the development of SDH [13,14]. In pediatric patients with chronic SDH and AC, symptoms include headache and vomiting, whereas hemiparesis was prevalent in patients without AC. Patients with chronic SDH and AC have been found to be significantly younger [11]. In a review of 658 patients with SDH or hygroma, AC of the MCF was found in 2.43% of patients [15]. The pediatric age group makes up 30%-42% of AC chronic SDH patients, with up to 86% of these patients having the AC in the MCF [11,16]. Bilateral presentation of chronic SDH is seen in up to 6% [16]. A theory for the susceptibility to develop hemorrhage is based on the decreased compliance properties of the AC [13,16]. It is suspected that veins between the dura and the AC contribute to the hemorrhage [16-18]. Management of AC associated with SDH has been overwhelmingly operative as reported in the literature [3,9,10,16,19]. Treatment options include craniotomy for early hemorrhages or burr holes for more chronic hemorrhages [12]. In patients with SDH and AC, there is no clear consensus regarding treatment of the AC at the time of SDH evacuation [15,16]. Conservative management was chosen in a minority with consideration of patient preference and a favorable clinical picture [16,19].

Our patient had a known Galassi Type III MCF AC which subsequently developed bilateral SDH. On MRI, CSF signal of the known right MCF AC was replaced by hypointense T1 weighted signal consistent with intra-cyst hemorrhage in continuity to the right SDH. The contralateral hemorrhage demonstrated intensity and density consistent with acute hemorrhage on MRI and CT, respectively. Without a significant history of recent trauma to explain an acute hemorrhage, a possible explanation for the left-sided contralateral hemorrhage would be decreased intracranial pressure (ICP) resulting from AC rupture. Findings supporting this included signs of decreased ICP as represented by the diffuse pachymeningeal enhancement seen on his follow-up MRI. The pachymeningeal enhancement likely represents engorgement of the dura with blood that occurs with decreased CSF pressure [20]. As the intracranial compliance increased after cyst rupture, ICP is suggested to have decreased, producing an intracranial hypotension effect. We propose that cyst rupture and ipsilateral formation of hemorrhage occurred first. The subsequent intracranial hypotension is the theorized mechanism by which the left SDH developed in this patient. Our patient did not have significant clinical or imaging findings suggesting significantly increased ICP. Given adequate pain control, and a neurological exam that could be trended, the decision for conservative management was made. This strategy was successful and is a departure from the overwhelming operative management strategies found in the literature.

## Conclusions

AC is a known risk factor for the development of SDH in young male patients after trivial trauma. Surgical evacuation in AC patients with SDH is considered the appropriate management strategy in the vast majority of patients. We show that conservative management with serial imaging may be obtained with appropriate clinical follow up if the patient is asymptomatic/mildly symptomatic and neurologically intact. This, therefore, avoids the need for surgery. In this case of non-operative management in bilateral SDH associated with AC, we propose that the development of intracranial hypotension secondary to AC rupture contributed to the development of his bilateral SDH.
